# The Potential Protective Effect and Underlying Mechanisms of Physiological Unconjugated Hyperbilirubinemia Mediated by UGT1A1 Antisense Oligonucleotide Therapy in a Mouse Model of Cyclosporine A-Induced Chronic Kidney Disease

**DOI:** 10.3390/metabo12100999

**Published:** 2022-10-20

**Authors:** Basma H. Marghani, Mohamed El-Adl, Ahmed I. Ateya, Basma H. Othman, Heba I. Ghamry, Mustafa Shukry, Mohamed Mohamed Soliman, Mohamed Abdo Rizk

**Affiliations:** 1 Department of Physiology, Faculty of Veterinary Medicine, Mansoura University, Mansoura 35516, Egypt; 2 Department of Biochemistry, Physiology, and Pharmacology, Faculty of Veterinary Medicine, King Salman International University, South of Sinaa 46612, Egypt; 3 Department of Biochemistry, Faculty of Veterinary Medicine, Mansoura University, Mansoura 35516, Egypt; 4 Department of Husbandry & Development of Animal Wealth, Faculty of Veterinary Medicine, Mansoura University, Mansoura 35516, Egypt; 5 Medical Research Center, Faculty of Medicine, Mansoura University, Mansoura 35516, Egypt; 6 Department of Home Economics, College of Home Economics, King Khalid University, P.O. Box 960, Abha 61421, Saudi Arabia; 7 Department of Physiology, Faculty of Veterinary Medicine, Kafrelsheikh University, Kafrelsheikh 33516, Egypt; 8 Department of Biochemistry, Faculty of Veterinary Medicine, Benha University, Benha 13518, Egypt; 9 Department of Internal Medicine and Infectious Diseases, Faculty of Veterinary Medicine, Mansoura University, Mansoura 35516, Egypt

**Keywords:** cyclosporine A, chronic kidney disease, oxidative stress, UGT1A1 antisense, mild unconjugated hyperbilirubinemia

## Abstract

Cyclosporine A (CSA) is an immunosuppressive drug that has improved transplant survival rates. However, its use is often limited because it is thought to be linked to the development of chronic kidney disease after kidney transplants. This study aimed to investigate the protective effects and underlying mechanisms of physiological unconjugated (UC) hyperbilirubinemia mediated by UGT1A1 antisense oligonucleotide in a mouse model of CsA-induced chronic kidney disease, and match these with that of chitosan (CH) as a natural chelator against kidney injury. In the current study, CsA-treated mice were given an intravenous injection of UGT1A1 antisense morpholino oligonucleotide (16 µg/kg) every third day for 14 days. In serum samples, bilirubin, creatinine, and urea were determined. Markers of oxidative stress, antioxidant activities, and mRNA expression of target genes PPAR-α, cFn, eNOS, NF-B, AT1-R, ETA-R, Kim-1, and NGAL were measured in the kidney tissues. Moreover, histopathological examinations were carried out on the kidney tissue. Physiological UC hyperbilirubinemia could be a promising protective strategy against CsA-induced kidney disease in transplant recipients. UGT1A1 antisense oligonucleotide-induced physiological UC hyperbilirubinemia serum significantly protected against CsA-induced kidney dysfunction. UCB acts as a signaling molecule that protects against kidney disease through different mechanisms, including antioxidant, anti-inflammatory, and hormonal action, by activating nuclear hormone receptors (PPAR-α). Moreover, it significantly downregulated mRNA expression of NF-kB, ETA-R, iNOS, AT1-R, cFn, Kim-1, and NGAL in the kidney tissue and alleviated CsA-induced kidney histological changes in CsA-treated mice.

## 1. Introduction

Globally, chronic kidney disease (CKD) is increasing and is associated with a higher risk of premature death [1], which is characterized by oxidative stress, vascular inflammation, and endothelial dysfunction [2]. Despite advances in medical treatment over the previous decades, clinical outcomes in patients with severe CKD, particularly those with end-stage renal disease (ESRD), have not significantly improved [3]. ESRD is treated with hemodialysis, peritoneal dialysis, or a kidney transplant [4]. Kidney transplantation enhances a patient’s daily activities and results in a higher survival rate; however, organ rejection is possible [5]. The incidence of autoimmune diseases has resulted in the effective use of immunosuppressive agents to achieve better clinical outcomes [6].

Cyclosporine A (CsA), a well-known calcineurin inhibitor with immunosuppressive properties, is one such agent that has been shown to prevent graft rejection after transplantation and provide symptomatic relief in a wide range of autoimmune diseases [7]. However, the long-term use of CsA causes side effects such as nephrotoxicity [8]. The effects of CsA therapy on nephrotoxicity, kidney lesions, and creatinine levels have been previously reported [9]. CsA causes an increase in reactive oxygen species (ROS) and lipid peroxidation products, both of which have been linked to its toxicity [10]. CsA causes kidney damage, accompanied by an increase in inflammatory and oxidative stress markers [11]. The importance of inflammation and altered oxidative homeostasis in the pathogenesis of CsA-induced kidney dysfunction is further supported by studies conducted in experimental models, where antioxidant and anti-inflammatory therapeutic agents have been shown to protect CKD [12].

Recently, bilirubin’s potential mechanisms of action in preventing CKD progression and renal transplant rejection have been investigated [13]. Bilirubin’s antioxidant and anti-inflammatory properties can be attributed to its preventive effects [14]. Bilirubin is produced by the breakdown of heme, derived primarily from red blood cell hemoglobin or other hemoproteins such as myoglobin, cytochromes, and catalase [15]. Heme is broken down in heme catabolism by heme oxygenase (HO), which produces biliverdin, which is then converted into bilirubin by biliverdin reductase [16]. In the blood, bilirubin binds to albumin and is carried to the liver, where it is conjugated with glucuronic acid by uridine diphosphate-glucuronyl transferase 1A1 (UGT1A1) and discharged into bile via multi-drug resistance protein-2 [17]. Bilirubin has a crucial role in human physiology as higher serum bilirubin concentrations in physiological ranges (1–5 mg/dL) are associated with a lower risk of hypertension, obesity, diabetes mellitus, development and progression of chronic kidney disease (CKD), and all-cause mortality [18].

Unconjugated bilirubin (UCB), a metabolite of heme catabolism, is dangerous when raised above the laboratory reference range, due to diminished conjugation that leads to decreased bilirubin plasma clearance and jaundice [19]. However, recent studies have shown that moderately elevated serum UCB levels have potent antioxidant effects [19]. UCB has radical scavenging activity, which protects proteins and lipids from oxidation. It effectively inhibits amino acid oxidation mediated by hydroxyl, hydroperoxyl, and superoxide radicals produced by radiolysis [20] and irradiation [21]. UCB is also an effective antioxidant, scavenging synthetic peroxyl radicals [22] and inhibiting copper-induced lipid oxidation [23]. Mild unconjugated hyperbilirubinemia in mice was induced by using specific morpholino antisense oligonucleotides to target hepatic UGT1A1, the enzyme responsible for bilirubin conjugation in the liver [24]. Intravenous injections of UGT1A1 antisense morpholinos every 72 h resulted in hepatic UGT1A1 being knocked down in vivo, 54% decrease in UGT1A1 protein levels in the liver, and a three-fold increase in unconjugated plasma bilirubin levels [24].

Subsequently, the current study aimed to investigate whether physiological unconjugated hyperbilirubinemia induced by UGT1A1 antisense morpholinos protects against the progression of CKD in a mice model of cyclosporine A-induced nephropathy. The reno-protective effects of endogenously elevated UCB were comparable with that of chitosan (CH), a natural chelator against renal toxicity [25], and a free radical scavenging pharmacologic material [26].

## 2. Materials and Methods

### 2.1. Drugs and Reagents

Cyclosporine A (CsA) was provided by Novartis Pharmaceuticals (Sandimmun Neoral 50 mg soft gelatin capsules; Novartis, Bâle, Switzerland). Neoral^®^ is an oral formulation of CsA. Chitosan (CH) was provided by Sigma-Aldrich (Sigma, St. Louis, MO, USA). UGT1A1 morpholino antisense oligonucleotides were obtained from Willow Fort Company, Birmingham, UK. All other reagents were of analytical grade.

### 2.2. Animals

Forty-eight male C57BL/6J mice (10–12 weeks old) weighing 30 ± 2 g were used in the present study and were purchased from the Medical Experimental Research Center (MERC), Faculty of Medicine, Mansoura University, Egypt. They were housed in a polypropylene cage in the animal house at MERC and allowed access to food and water ad libitum throughout the duration of the study. They were kept under standard conditions of 22 °C ± 2 °C with 12/12 h of light/darkness and 41–55% relative humidity. Animals were acclimatized to laboratory conditions for one week before the beginning of the study. The study protocol was performed according to the Guide for the Care and Use of Laboratory Animals. The Animal Ethical Research Committee approved all study procedures, Faculty of Veterinary Medicine, Mansoura University, (Code No. R/59).

### 2.3. Experimental Design and Treatment

Mice were randomly divided into six different groups (*n* = 8/group). Group I: Vehicle-treated group (control) received 0.9% NaCl, 1 mL/mice/day (i.v.) and olive oil, 1 mL/mice/day (p.o.). Group II: Chitosan-treated group (CH) received 0.9% NaCl, 1 mL/mice/day (i.v.) and(CH, 250 mg/kg/day (p.o.) [27]. Group III: UGT1A1 antisense morpholino-treated group (UGT1A1) received UGT1A1 antisense (5′-GCTCCAGCACACCACAGTCATGGT-3′, NCBI, 16 µg/kg/every third day, i.v.) [24]. Group IV: Cyclosporine A-treated group (CsA) received 0.9% NaCl, 1 mL/mice/day (i.v.) and CsA, 50 mg/kg/day dissolved in olive oil (p.o.) [28]. Group V: CsA + CH-treated group (CsA + CH) received CsA and CH identical to CsA and CH-treated groups. Group VI: CsA + UGT1A1-treated group (CsA + UGT1A1) received CsA and UGT1A1 antisense morpholino identical to the CsA and UGT1A1 antisense-treated groups. All treatments were administered for 14 days, and UGT1A1 antisense morpholino was given three days before the start of the study [29]. CsA was given to the animals one hour before CH oral gavage and UGT1A1 antisense morpholino by i.v. injection to evaluate their potential therapeutic effects and reduce animal stress.

### 2.4. Blood and Tissue Harvesting

After treatment for 14 days, all mice were fasted overnight and humanely sacrificed. Blood was harvested by cardiac puncture, and the serum was separated for assaying total bilirubin (TB), direct conjugated bilirubin (CB), creatinine, and urea. Kidneys were dissected and rinsed in physiological saline. One dissected kidney was frozen at −80 °C for assays of oxidative stress markers and enzymatic antioxidant activities. The other kidney was sliced and either snap-frozen in liquid nitrogen for mRNA gene expression or fixed in 4% buffered formalin for histological analysis. 

### 2.5. Tissue Processing

Kidney tissue homogenate was prepared by homogenizing 500 mg of kidney tissue in 5 mL phosphate buffer solution (0.01 M sodium phosphate buffer, pH 7.4, containing 0.14 M NaCl). Homogenates were centrifuged at 3500 rpm for 10 min. The supernatant was collected and used for biochemical assays of malondialdehyde (MDA), a lipid peroxidation marker, and nitric oxide (NO), an oxidative stress marker. The enzymatic antioxidants reduced glutathione (GSH), glutathione S-transferase (GST), catalase (CAT), glutathione peroxidase (GPx), and superoxide dismutase (SOD).

### 2.6. Serum Bilirubin Analysis

Total and direct bilirubin colorimetric assay kits (Wako Diagnostics, Mountain View, CA, USA) were used for the determination of total bilirubin (TB) and direct bilirubin (CB), following the instructions of the manufacturer [30]. The difference between total bilirubin and conjugated bilirubin was used to calculate indirect bilirubin (UCB) [28].

### 2.7. Serum Creatinine and Urea Analysis

According to the diagnostic kit manual, creatinine and urea were determined in serum spectrophotometrically using commercial kits (Gamma Trade Co, Cairo, Egypt).

### 2.8. Tissue Oxidative Stress Markers and Enzymatic Antioxidant Analysis

Kidney tissue homogenates were used to determine malondialdehyde (MDA) oxidative stress markers. Nitric oxide (NO), as well as enzymatic antioxidants including reduced glutathione (GSH), glutathione S-transferase (GST), catalase (CAT), glutathione peroxidase (GPx), and superoxide dismutase (SOD), were from commercial kits (Bio Diagnostic Co., Giza, Egypt), used according to the manufacturer’s instructions. The MDA assay was based on thiobarbituric acid reacting with MDA to create thiobarbituric acid reactive species (pink-colored products) that were calorimetrically detected at 534 nm relative to MDA content. Nitric oxide (NO), an oxidative stress marker, was calculated based on the formation of nitrous acid diazotise sulphanilamide in the presence of nitrite, and the product being coupled with N-(1–naphthyl) ethylenediamine. The resulting azo dye has a bright reddish-purple color, which can be measured at 540 nm. GSH activity was determined based on the reaction of 5,5 dithiobis 2-nitrobenzoic acid (DTNB) with glutathione, where a relatively stable yellow color was produced and spectrophotometrically measured at 412 nm. GST activity was assayed based on the formation of GS-DNB, creating a dinitrophenyl thioether that can be spectrophotometrically detected at 340 nm. CAT activity was determined based on its reaction with a known quantity of hydrogen peroxide (H_2_O_2_) and was calorimetrically measured at 510 nm. GPx activity was estimated based on the conversion of NADPH to NADP+. Absorbance was analyzed at 340 nm. SOD activity was assayed based on its capacity to prevent the phenazine methosulphate-mediated reduction of nitro blue tetrazolium dye.

### 2.9. RNA Extraction and Reverse Transcription

Total RNA was isolated from snap-frozen kidneys using TRIzol reagent (TransGen Biotech, Beijing, China) and the Direct-zol^TM^ RNA MiniPrep extraction kit, following the manufacturer’s protocol. The purity and concentration of total RNA samples were measured by a nanodrop (UV-Vis spectrophotometer Q5000/USA). The cDNA synthesis was performed with 1 μg of total RNA using the SensiFast^TM^ cDNA synthesis kit. The whole reaction mixture was carried out in a volume of 20 μL, consisting of 1 μg RNA, 4 μL buffer, 1 μL reverse transcriptase, and up to 20 μL DNase-free water. The thermal cycler protocol was 25 °C primer annealing for 10 min, 42 °C reverse transcriptions for 15 min, 85 °C inactivations for 5 min, and a final hold at 4 °C. All laboratory operations followed the guidelines from the Veterinary Personal Biosecurity & Infection Control Handbook for biosecurity and infection control [31].

### 2.10. Quantitative Real-Rime PCR (qRT-PCR) Analysis

SYBR Green PCR Master Mix (2× SensiFast^TM^ SYBR, Bioline, Essex, UK) was used to measure mRNA levels of PPAR, cFn, eNOS, NF-B, AT1-R, and ETA-R genes in tissues. The overall reaction mixture were20 μL, with two 10 μL × SensiFast SYBR, 2 μL cDNA, 6.4 μL H_2_O (d.d. water), and 0.8 μL of each primer. A melting curve was created for the specificity of the PCR product confirmation. Sangon Biotechnology (Shanghai, China) synthesized the PCR primers, which are shown in (Supplementary Table S1). The data were processed with SPSS 19, and the 2^−ΔΔCt^ technique was used to calculate the mRNA expression of each gene per sample, with GAPDH as the control.

### 2.11. Histological Examination and Quantitative Analysis of Renal Tissue Lesions

Kidney paraffin sections (5 μm thickness) were cut and stained according to the standard hematoxylin and eosin (H&E) staining protocol using the H&E Staining kit (Beyotime Institute of Biotechnology, Nantong, China; cat no. C0105). Selected slides from each group were also stained with special stains, using Masson’s trichrome staining kit (cat no. ab150686) and periodic acid–Schiff staining kit (cat no. ab150680). All microscopic slides were examined at 400× magnification on a light microscope and microscopic images were taken. 

#### Quantitative Analysis of Renal Tissue Lesions

Quantitative stained sections were analyzed using the Image J software program (NIH, version 1.36, National Institutes of Health, Bethesda, MD, USA). H&E-stained sections were examined for histological alterations and semiquantitatively scored for tubular dilatation, tubular degeneration, and tubular necrosis. The degree of tubular damage was graded from 0 to 5, according to Houghton et al. [32] Depending on severity, 0 = no renal lesion; 1 = minimal renal lesion of the cortex and medulla (<1%); 2 = mild renal lesion of the cortex and medulla (1–25%); 3 = moderate renal lesion of the cortex and medulla (26–50%); 4 = moderate renal lesion of the cortex and medulla (51–75%); and 5 = severe/high renal lesions of the cortex and medulla (76–100%). In Masson’s trichrome staining, collagen fibers and fibrosis were represented by the blue staining area. According to Oruc et al. [33], renal interstitial fibrosis was assessed in Masson trichrome stained slides by calculating the percentage of regions with interstitial fibrosis per cortex field in three fields from each slice. The given scores were graded from 0 to 5: 0 for normal interstitial, 1 for <10% of areas injured, 2 for 11–25% of areas injured, 3 for 26–50% of areas injured, 4 for 51–75% of areas injured, and 5 for >76% of areas injured. PAS staining illustrates the precipitated glycogen in the basement membrane and assesses the percentage of renal tubular injury. In PAS-stained slides, the following glomerular sclerosis grades were assigned: 0 representing no lesions, 1 representing 1–25%, 2 representing 25–50%, 3 representing 50–75%, and 4 representing 75–100%, according to Li et al. [34], All data obtained by each mouse were statistically analyzed.

### 2.12. Statistical Analysis

The statistical package for social sciences (SPSS) program was used to analyze the results (version 19, IBM Analytics, New York, NY, USA). All values were presented as mean ± standard error (SE). For multiple comparisons, data were analyzed using a one-way analysis of variance (ANOVA), followed by Duncan’s multiple range comparison (DMTs) post hoc analysis tests. Differences were considered statistically significant at *p* < 0.05. 

## 3. Results

### 3.1. UGT1A1 Antisense Oligonucleotide Mediates Physiological Unconjugated Hyperbilirubinemia in CsA-Treated Mice

**Figure 1:**
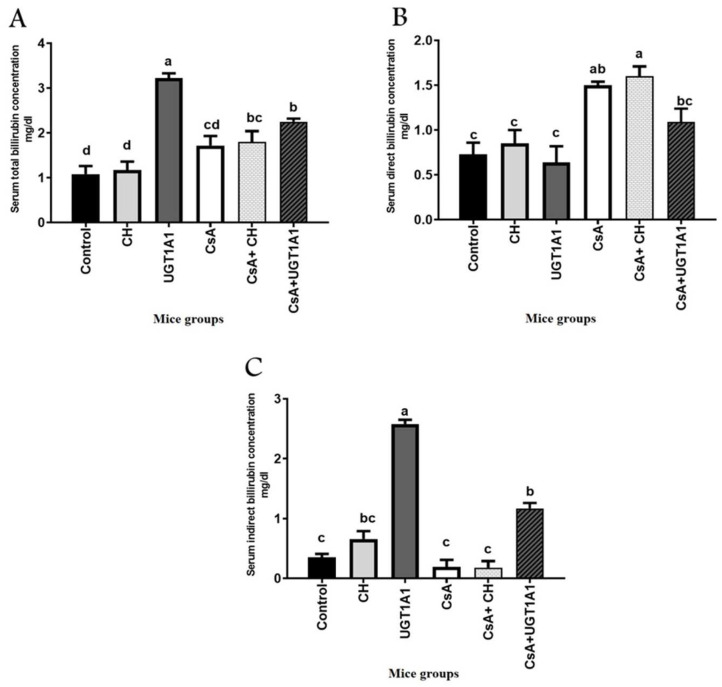
UGT1A1 antisense oligonucleotide mediates physiological unconjugated hyperbilirubinemia in CsA-treated mice: (**A**) total bilirubin (TB); (**B**) direct bilirubin (conjugated bilirubin; CB); and (**C**) indirect bilirubin (unconjugated bilirubin; UCB). Values are expressed as mean ± SEM (*n* = 8). Means of different letters are statistically different (*p* < 0.05) (ANOVA; Duncan’s post hoc analysis).

Physiological hyperbilirubinemia was assessed by measuring serum bilirubin concentrations in mice treated with CH, UGT1A1 antisense oligonucleotide, CsA, CsA + CH, and CsA + UGT1A1 antisense oligonucleotide (Figure 1). The CH-treated group had non-significant increases in serum total bilirubin, direct bilirubin (CB), and indirect bilirubin (UCB) concentrations compared with the control group. Meanwhile, the group treated with UGT1A1 antisense oligonucleotide showed a significant (*p* < 0.05) increase in serum total bilirubin (Figure 1A), a significant decrease in direct bilirubin (Figure 1B), and a small but significant (*p* < 0.05) increase in serum indirect bilirubin levels compared with the control group (2.85 ± 0.073 vs. 0.35 ± 0.07) and CH-treated group (2.85 ± 0.073 vs. 0.66 ± 0.14). The CsA-treated group showed a significant (*p* < 0.05) decrease in serum total bilirubin (Figure 1A), increase in direct bilirubin levels (Figure 1B), and significant decrease in indirect bilirubin levels (Figure 1C) compared with the UGT1A1 antisense oligonucleotide-treated group, though it was not significantly different from the control or CH-treated groups. However, the group treated with UGT1A1 antisense in combination with CsA had a significant (*p* < 0.05) small increase in serum UCB within physiological ranges (physiological UC hyperbilirubinemia) compared with the CsA-treated group (1.17 ± 0.09 vs. 0.198 ± 0.12) and CsA + CH-treated group (1.17 ± 0.09 vs. 0.1825 ± 0.14) (Figure 1C). 

### 3.2. Physiological Unconjugated Hyperbilirubinemia Protects against CsA-Induced Kidney Dysfunction

**Figure 2:**
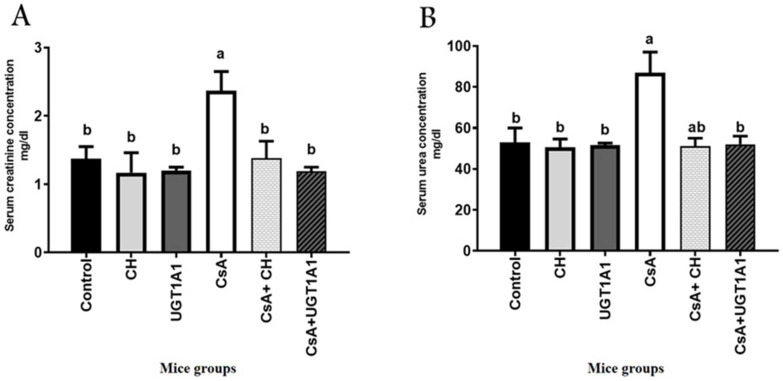
Physiological unconjugated hyperbilirubinemia protects against CsA-induced kidney dysfunction: (**A**) creatinine and (**B**) urea (U). Values are expressed as mean ± SEM (*n* = 8). Means of different letters are statistically different (*p* < 0.05) (ANOVA; Duncan’s post hoc analysis).

Induction of physiological UC hyperbilirubinemia by UGT1A1 antisense oligonucleotide protects against CsA-induced kidney dysfunction such as that observed with the reference antioxidant and natural chelator, chitosan (Figure 2A,B). The severity of CsA-induced kidney dysfunction was determined by measuring serum creatinine and urea levels. The creatinine and urea levels of mice treated with CsA are shown in Figure 2A,B. The CsA-treated group had significant (*p* < 0.05) increases in serum creatinine and urea levels compared with the control group (Figure 2A,B). Compared with the CsA-treated group, the CsA + CH group had a significant (*p* < 0.05) decrease in serum creatinine levels (Figure 2A). Nevertheless, the group treated with UGT1A1 antisense oligonucleotides in combination with CsA showed a significant (*p* < 0.05) reduction in both creatinine and urea levels compared with the CsA-treated group.

### 3.3. Physiological Unconjugated Hyperbilirubinemia Inhibits CsA-Induced Kidney Oxidative Stress

**Figure 3:**
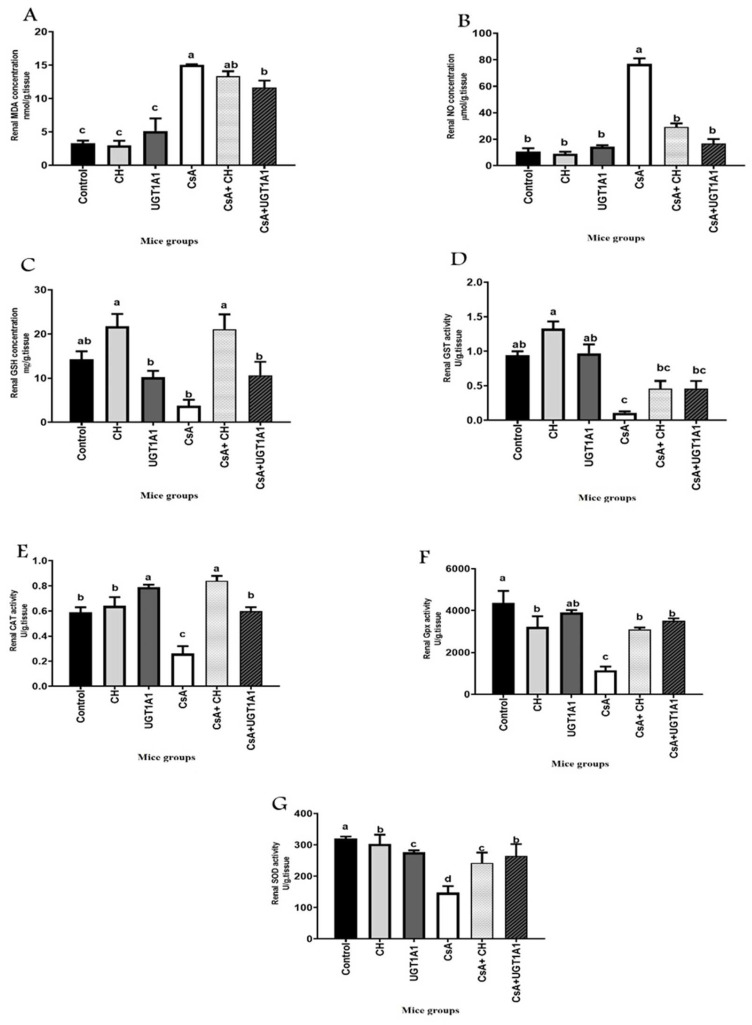
Physiological unconjugated hyperbilirubinemia inhibits CsA-induced kidney oxidative stress: (**A**) malondialdehyde (MDA); (**B**) nitric oxide (NO); (**C**) reduced glutathione (GSH); (**D**) glutathione S-transferase (GST); (**E**) catalase (CAT); (**F**) glutathione peroxidase (GPx), and (**G**) superoxide dismutase (SOD). Values are expressed as mean ± SEM (*n* = 8). Means of different letters are statistically different (*p* < 0.05) (ANOVA; Duncan’s post hoc analysis).

Oxidative damage, particularly to lipids, is associated with the progression and severity of CKD. The antioxidant effects of physiological unconjugated hyperbilirubinemia on CsA-induced oxidative stress in the kidney tissues of mice were examined (Figure 3). The CsA-treated group demonstrated higher renal oxidative stress, as evidenced by significant (*p* < 0.05) increases in oxidative stress markers that included malondialdehyde (MDA), a lipid peroxidation marker, and nitric oxide (NO) concentrations (Figure 3A,B), and significant (*p* < 0.05) decreases in antioxidant enzymes glutathione-S transferase (GST), catalase (CAT), glutathione peroxidase (GPx), and superoxide dismutase (SOD) activity compared with the control group (Figure 3D–G). UGT1A1 antisense oligonucleotides alone induced a substantial (*p* < 0.05) increase in CAT activity (Figure 3E) compared with the control group. Treatments with CH or UGT1A1 antisense morpholino, in combination with CsA, inhibited CsA-induced kidney oxidative stress. UGT1A1 antisense was more effective, causing significant (*p* < 0.05) decreases in levels of oxidative stress markers MDA and NO levels (Figure 3A,B), as well as significant increases in antioxidant GPx and SOD activity (Figure 3F,G) when compared with the CsA-treated group.

### 3.4. Unconjugated Bilirubin, a Signaling Molecule, Activates PPAR-α Gene Expression in CsA-Treated Mice

**Figure 4:**
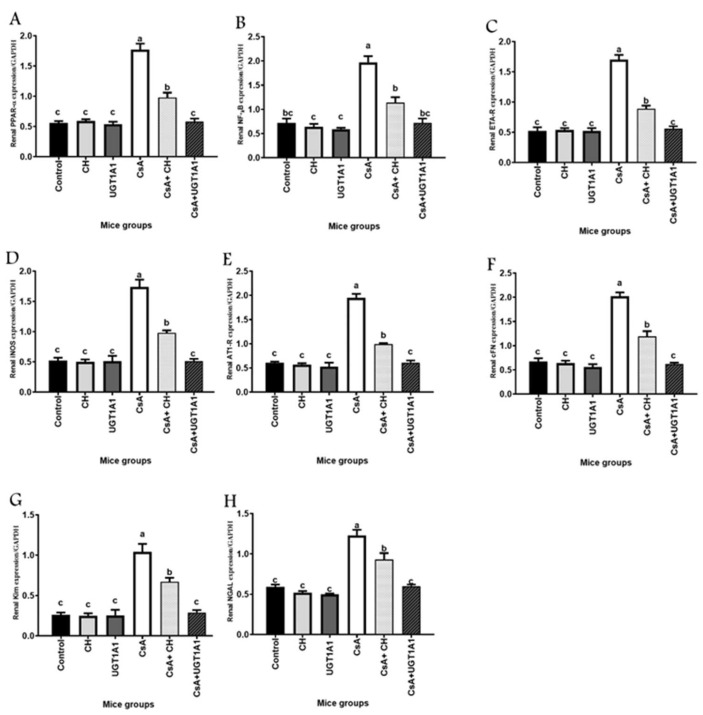
Unconjugated bilirubin, a signaling molecule, activates PPAR-α and regulates NF-kB, ETA-R, iNOS, AT1-R, cFn, Kim-1, and NGAL gene expression in CsA-treated mice: (**A**) peroxisome proliferator-activated receptor alpha (PPAR-α); (**B**) nuclear factor kappa B (NF-kB); (**C**) endothelin type A-receptor (ETA-R); (**D**) inducible nitric oxide synthase (iNOS); (**E**) angiotensin type1-receptor (AT1-R); (**F**) cellular fibronectin (cFN); (**G**) kidney injury molecule-1 (Kim-1); and (**H**) neutrophil gelatinase-associated lipocalin (NGAL). Values are expressed as mean ± SEM (*n* = 8). Means of different letters are statistically different (*p* < 0.05). (ANOVA; Duncan’s post hoc analysis).

As shown in Figure 4, CsA significantly (*p* < 0.05) reduces the relative expression of peroxisome proliferator-activated receptor alpha (PPAR-α) mRNA compared with the control group. Meanwhile, combining CH and/or UGT1A1 antisense oligonucleotide with CsA significantly (*p* < 0.05) increased PPAR-α mRNA expression in the kidney tissues of CsA + CH and CsA + UGT1A1 mice groups. Hepatic UGT1A1 antisense induced physiological unconjugated hyperbilirubinemia, a metabolic signaling molecule that activates PPAR-α, which regulates many physiological transcription factors and protects against CsA-induced kidney injury such as the reference natural chelator, chitosan (Figure 4A). 

### 3.5. Unconjugated Bilirubin, a Signaling Molecule, Regulates NF-kB, ETA-R, iNOS, AT1-R, cFn, Kim-1, and NGAL Gene Expression in CsA-Treated Mice

CsA-induced inflammation, vascular resistance, nitric oxide production, vasopressor, fibrosis, and kidney injury were mediated by significant (*p* < 0.05) increases in the expression of nuclear factor kappa B (NF-kB), endothelin type A-receptor (ETA-R), inducible nitric oxide synthase (iNOS), angiotensin type 1-receptor (AT1-R), cellular fibronectin (cFN), kidney injury molecule-1 (Kim-1), and neutrophil gelatinase-associated lipocalin (NGAL) compared with the control group (Figure 4B–H). In comparison to the CsA-treated group, CsA + CH and/or CsA + UGT1A1 antisense significantly (*p* < 0.05) regulated NF-kB, ETA-R, iNOS, AT1-R, and cFn genes, as well as Kim-1 and NGAL (Figure 4B–H). Our findings revealed that physiological unconjugated hyperbilirubinemia had a marked effect on renal gene expression of the target genes compared with that of the natural chelator, chitosan. 

### 3.6. Physiological UC Hyperbilirubinemia Alleviates CsA-Induced Histological Changes in the Kidney 

#### 3.6.1. Hematoxylin and Eosin-Stained Kidney Sections

**Figure 5:**
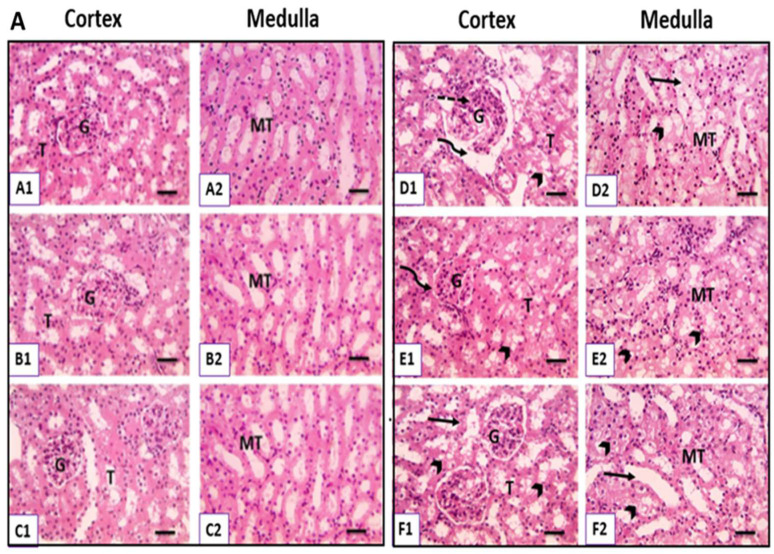

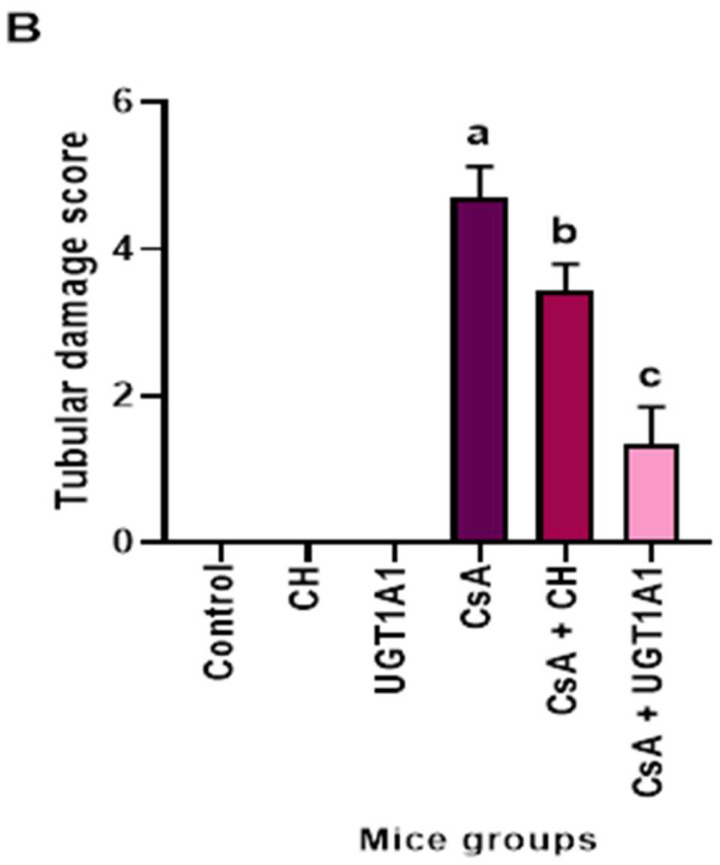
(**A**) Photomicrograph of kidney sections in different experimental groups (H&E; X: 400). Normal histological structure of cortical glomeruli (G) and tubules (T) beside normal medullary tubules (MT) in kidney sections of the control group (**A1**,**A2**), CH-treated group (**B1**,**B2**), and UGT1A1 antisense oligonucleotide-treated group (**C1**,**C2**). Glomerular hypertrophy dilated Bowman’s space (curved arrow) with mesangial proliferation (dashed arrows), tubular degeneration in the cortex (arrowheads), tubular degeneration (arrowheads), and necrosis (black arrows) in the medulla in kidney sections of the CsA-treated group (**D1**,**D2**). Narrowed Bowman’s space (curved arrow), mild to moderate tubular degeneration in the cortex (arrowheads), and tubular degeneration in the medulla (arrowheads) in kidney sections of the CsA + CH treated group (**E1**,**E2**). Very mild tubular dilation (black arrows) and degeneration (arrowheads) in cortex and medulla in kidney sections of the CsA + UGT1A1 antisense oligonucleotide-treated group (**F1**,**F2**). (**B**) Semiquantitative analysis of renal tissue lesions based on tubular damage score in different experimental groups. Values are expressed as mean ± SEM (*n* = 8). Means of different letters are statistically different (*p* < 0.05). (ANOVA; Duncan’s post hoc analysis).

Microscopic pictures of H&E-stained kidney sections are shown in Figure 5A. Kidneys of the control group (Figure 5A1,A2), CH-treated group (Figure 5B1,B2), and UGT1A1 antisense oligonucleotide (Figure 5C1,C2) showed the normal histological structure of cortical glomeruli (G) and tubules (T) besides normal medullary tubules (MT). In contrast, kidney sections from the CsA-treated group revealed marked histological changes, including glomerular hypertrophy, dilated Bowman’s space with mesangial proliferation, tubular degeneration in the cortex, tubular degeneration, and necrosis in the medulla (Figure 5D1,D2). However, kidney sections from the CsA + CH-treated group showed a narrowed Bowman’s space, mild to moderate tubular degeneration in the cortex, and tubular degeneration in the medulla (Figure 5E1,E2). At the same time, kidney sections from the CsA + UGT1A1 antisense oligonucleotide-treated group exhibited very mild tubular dilation and degeneration in the cortex and medulla (Figure 5F1,F2). The quantitative analysis of renal tissue lesions based on tubular damage in different experimental groups revealed that the CsA-treated group had the highest tubular damage score, as shown in Figure 5B. The tubular damage score was significantly reduced when CH and UGT1A1 antisense oligonucleotides were given after CsA. However, UGT1A1 antisense oligonucleotide-mediated physiological unconjugated hyperbilirubinemia was more effective in decreasing the severity of tubular damage and alleviating renal histological changes.

#### 3.6.2. Masson Trichrome-Stained Kidney Sections

Microscopic pictures of Masson trichrome-stained kidney sections are shown in Figure S1A. Kidneys of the control group (Supplementary Figure S1A1,A2), CH-treated group (Supplementary Figure S1B1,B2), and UGT1A1 antisense oligonucleotide-treated group (Supplementary Figure S1C1,C2) showed no collagen deposition in the interstitial tissue in the cortex nor the medulla. In contrast, kidney sections from the CsA-treated group revealed excessive, bluish-stained collagen deposition in the interstitial tissue of the cortex and medulla (Supplementary Figure S1D1,D2). On the other hand, kidney sections from the CsA + CH-treated group showed markedly decreased bluish-stained collagen deposition in interstitial tissue of the cortex and medulla (Supplementary Figure S1E1,E2). In comparison, kidney sections from the CsA + UGT1A1 antisense oligonucleotide-treated group exhibited moderately decreased bluish-stained collagen deposition in interstitial tissue in the cortex and medulla (Supplementary Figure S1F1,F2). UGT1A1 antisense oligonucleotides, combined with CsA, significantly induced a mild renal fibrosis score compared with CsA-treated mice. As noted in Supplementary Figure S1B, quantitative analyses of renal tissue lesions based on the renal fibrosis scores of the different experimental groups showed that the CsA-treated group had a severe interstitial fibrosis score.

#### 3.6.3. PAS-Stained Kidney Sections

Microscopic pictures of PAS-stained kidney sections are shown in Figure S2. Kidneys of the control group (Supplementary Figure S2A1,A2), CH-treated group (Supplementary Figure S2B1,B2), and UGT1A1 antisense oligonucleotide-treated group (Supplementary Figure S2C1,C2) shows normal structure of cortical glomeruli and tubules beside normal medullary tubules. Kidney sections from the CsA-treated group revealed glomerular swelling and increased thickness of PAS-positive glomerular basement membrane with markedly increased renal capsular space and absent MT brush borders (Supplementary Figure S2D1,D2). Kidney sections from the CsA + CH-treated group showed markedly decreased renal capsular space with retained PAS-positive MT brush borders (Supplementary Figure S2E1,E2). However, kidney sections from the CsA + UGT1A1 antisense oligonucleotide-treated group exhibited a mildly thickened glomerular basement membrane and partially retained PAS-positive MT brush borders (Supplementary Figure S2F1,F2). The UGT1A1 antisense oligonucleotide in combination with CsA significantly ameliorated renal sclerosis compared with the CsA-treated mice group. The quantitative analysis of renal tissue lesions based on the renal sclerosis scores of the different experimental groups revealed that the CsA-treated group had the highest glomerulosclerosis score, as shown in Supplementary Figure S2B.

## 4. Discussion

Supplementation CsA is an immunosuppressive drug that has improved transplant survival rates [7]. However, its use is often limited as a second-line agent because it is thought to be linked to the development of chronic allograft nephropathy following kidney transplant [35]. There is growing evidence that CsA therapy contributes to chronic renal failure [36] and impairs renal blood flow after kidney transplantation [37]. Alterations in redox homeostasis and chronic systemic inflammation are two primary mechanisms of CsA-induced organ damage [38]. Increased levels of unconjugated bilirubin (UCB) after renal transplant have been demonstrated to be a good predictor of graft survival, which may be connected to renal transplant acceptance [39]. Animals with moderate hyperbilirubinemia from partial knockdown of hepatic UGT1A1 showed improvements in glomerular filtration rate, renal blood flow, and renal vascular resistance [40]. As a result of the antioxidant and anti-inflammatory properties of UCB, the present study was carried out to evaluate the protective effect of a mild increase in UCB within the physiological range on the prevention of kidney damage and development of ESRD in a mice model of CsA-induced nephropathy. In the current study, we used morpholino antisense oligonucleotides to suppress hepatic UGT1A1 and induce mild hyperbilirubinemia in mice. Morpholino oligos differ significantly from natural nucleic acids in that methylene morpholine rings replace the ribose or deoxyribose sugar moieties and non-ionic phosphonodiamidite linkages replace the anionic phosphates of DNA and RNA. Each morpholine ring places one of the basic DNA bases (A, C, G, and T) for pairing, enabling a 25-base morpholino oligo to bind to its complementary 25-base target site in an RNA strand via Watson–Crick pairing. Due to its uncharged backbone, the morpholino oligo has many advantages over traditional antisense oligos or small interfering RNA (siRNA), including resistance to nucleases, which allows it to stay in vivo longer, and reduced “off-target” effects compared with traditional antisense DNA molecules with sulphur backbones [19].

Our findings show that CsA-treated mice had a moderate increase in blood total bilirubin with a significant rise in conjugated bilirubin and a substantial decrease in serum unconjugated bilirubin compared with the control CH-treated group and UGT1A1 morpholino-treated group. CsA has been shown to reduce bile flow in animal studies, which could explain why high doses cause hyperbilirubinemia [41]. Furthermore, it is rapidly metabolized in the liver and interacts with the cytochrome P450 system (CYP 3A4), making it vulnerable to severe drug–drug interactions [42]. In comparison, mice that were given UGT1A1 antisense morpholino oligonucleotide alone or in combination with CsA every third day had significantly higher serum UCB levels than CsA- and CsA + CH-treated mice, owing to increased total bilirubin and decreased conjugated bilirubin. Treatment with UGT1A1 antisense morpholinos can knock down hepatic UGT1A1, resulting in a significant reduction in UGT1A1 protein levels in the liver, which is linked to a considerable physiological increase in serum UCB [24].

The ability of the kidney tubules to maintain plasma urea and creatinine clearance reflects impaired kidney function [43]. CsA has been shown in animal studies to prolong the survival time of allogeneic organ transplantation and inhibit the cell-mediated immune response [44]. The primary reason for CsA’s limited clinical application is dose-dependent renal toxicity, which results in renal tubular atrophy, vacuolar degeneration, and renal failure [45]. In the current study, mice given CsA (50 mg/kg/d) for 14 days showed a significant (*p* < 0.05) increase in serum creatinine and urea levels compared with the control group, indicating kidney dysfunction. This finding is similar to what has been reported in CsA-treated rats [46]. Progressive deterioration of renal functions is the most common clinical manifestation of CsA treatment [47]. Furthermore, the impairment of arteriolar and glomerular vessels caused by CsA treatment have affected reabsorption efficiency and urea and uric acid excretions [48]. Nonetheless, our findings showed that CH treatment prevented kidney damage in CsA-treated mice, as evidenced by a significant (*p* < 0.05) reduction in creatinine compared with CsA-treated mice. Renal dialysis patients given CH for four weeks showed effective recovery of their renal functions compared with patients who did not receive supplements [49]. Furthermore, chitosan sulfate was protected against renal morphological and functional changes in glycerol-induced acute renal failure [50]. The specific distribution of LMWC to the kidneys [51] raises renal concentrations, enhancing the protective effect [52].

This is the first study to investigate the potential reno-protective effects of endogenous mild unconjugated hyperbilirubinemia caused by hepatic UGT1A1 inhibition in a mouse model of CsA-induced kidney damage. In the current study, the mouse group treated with UGT1A1 antisense morpholino, in combination with CsA, induced protection against kidney damage and improvement in renal functions, with a significant (*p* < 0.05) reduction in both creatinine and urea levels compared with the CsA group. Bilirubin administration significantly reduced kidney damage markers in rats with cyclosporine-induced nephropathy [34]. Mildly elevated serum bilirubin concentrations improved estimated glomerular filtration rate (eGFR) and lowered serum creatinine levels in patients with immunoglobulin A (IgA) nephropathy [51]. Many clinical studies show a link between circulating bilirubin levels and onset/progression of CKD [53]. Oda et al. [54] also found that patients with mild hyperbilirubinemia had a lower risk of developing end-stage kidney disease. UCB is a physiologically important factor that prevents glomerular dysfunction in individuals with hyperbilirubinemia [55].

Even in early CKD, increased oxidative stress is indicative of renal disease [56]. The level of oxidative damage is most prevalent in patients with ESRD [57]. This may lead to an increase in free radical generation, a loss in defenses against antioxidants, or both [1]. The primary mechanism implicated in CsA-induced kidney damage includes altered redox homeostasis [11]. CsA administration in the present study significantly increased oxidative stress markers MDA and NO and decreased the activity of enzymatic antioxidants GSH, CAT, GPx, and SOD in CsA-treated animals compared with the control group. This observation is consistent with previous research that has connected CsA cytotoxicity and oxidative state alterations [58]; in vivo and in vitro studies have established a relationship between CsA therapy and increases in ROS, MDA, and NO levels in kidney tissue [59]. Malondialdehyde (MDA) elevation is an essential in vivo lipid peroxidation marker [60]. CsA is considered a highly lipophilic agent that facilitates its attachment with organelle membranes, particularly the endoplasmic reticulum and mitochondria, making the cells more vulnerable to oxidative stress [61].

In the current study, UGT1A1 antisense was more effective. It restored redox homeostasis in kidney tissue, causing significant decreases in oxidative stress markers, MDA and NO, and significantly increased antioxidant enzymatic activity compared with the CsA-treated group. Bilirubin is a highly effective antioxidant that reduces oxidative damage [62]. Bilirubin is considered one of the best antioxidants for lipid peroxidation [63]. Exogenous UCB also reduced protein and lipid peroxidation levels [19]. Circulating markers of oxidative damage were enhanced in a hyperbilirubinemia Gunn rat model of adenine-induced renal failure [64]. Bilirubin was positively associated with GSH levels, which were higher in people with Gilbert’s syndrome compared with controls [65]. Therefore, elevated bilirubin in vivo increases the circulation capacity of antioxidants and can limit oxidative stress.

Many physiological pathways are disrupted in patients with CKD. Variations in the genes that control these pathways may influence the occurrence and susceptibility of this disease [66]. In this study, we investigated the effects of moderate unconjugated hyperbilirubinemia on the mRNA expressions of specific candidate genes linked to CKD progression in CsA-treated mice. Changes influence CsA-induced nephropathy in renal tissue gene expression [67]. In the current study, CsA therapy resulted in significant downregulation of relative expression of renal PPAR-α.

Meanwhile, relative NF-kB expression was significantly upregulated compared with the control group. Peroxisome proliferator-activated receptor-alpha (PPAR-α) is a nuclear hormone receptor transcription factor activated by ligands and regulates the expression of genes involved in inflammation and cellular lipid metabolism [68]. PPAR-α attenuated cisplatin-induced acute renal failure (ARF) in mice by blocking fatty acid (FAs) oxidation inhibition, lowering apoptosis and necrosis in the proximal tubule [69], and controlling inflammation by inhibiting the NF-_k_B pathway [70]. Activated PPAR-α has been shown to suppress the NF-_k_B pathway by inducing the inhibitory factor kB (IF_K_B) [71]. As a result, the increase in relative expressions of renal inflammatory mediator NF-_k_B in CsA-treated animals may be linked to a decrease in PPAR-α expression in this study. NF_k_B activation has been previously linked to the development of renal diseases [72]. Wang et al. [73] found that nephropathy is mediated via activation of the tumor necrosis factor/NF_k_B pathway As NF_k_B regulates inflammatory factor transcription in mesangial and tubular epithelial cells, it plays a vital role in the development and progression of renal diseases [74]. 

Treatment with CH or UGA1T1 antisense dramatically enhances PPAR-α mRNA expression while considerably decreasing relative expression of inflammatory marker NF_k_B in the CsA + CH and CsA + UGA1T1 groups compared with the CsA-treated group. Unconjugated bilirubinemia has recently been shown to be a nuclear receptor activator, specifically PPAR-α, and the structure of UCB revealed similarities to other known PPAR (peroxisome proliferator-activated receptor) activators [75]. Previous research has shown that PPAR induction protects renal hemodynamics in various models of renal insufficiency [76]. Blocking NF-_k_B expression reduces the production of reactive oxidative metabolites and increases kidney antioxidant capacity [77]. 

In the current study, relative expressions of endothelin one receptor-A (ETA-R) and inducible nitric oxide synthase (iNOS) in the kidney tissue of the CsA-treated group were significantly increased compared with the control group. Endothelin-1 (ET-1) and nitric oxide (NO) are implicated in CsA toxicity through interrelated pathways [78]. ET-1 is produced by endothelial and mesangial cells and is implicated in renal vascular resistance modulation in experimental models and humans [79]. It is involved in allograft vasculopathy and vasculitis [80]. Tubular cells generate ET-1 and express ETA-R isoforms in response to various stimuli [81]. Activation of ETR-A results in vasoconstriction and hypertension [82]. CsA caused apoptosis in multiple renal cell lines, mediated by NO via iNOS mRNA activation [83]. iNOS strongly increases the rate of NO production and oxidative stress [84]. In the present study, the upregulated expressions of both ETA-R and iNOS mRNA in kidney tissue contribute to a better understanding of the crosstalk between ETA-R and NO pathways in CsA nephropathy, as well as their role in the progression of CKD. Physiological unconjugated hyperbilirubinemia significantly decreased mRNA expression of vasoconstrictors ETA-R and iNOS in renal tissue, compared with the CsA-treated group. One of the first relevant biological functions of bilirubin is the ability to act as an antioxidant [85]. UCB reduces superoxide production and suppresses peroxynitrite formation [86]. Therefore, mild levels of UCB have the potential to be a beneficial disease-modifying agent in CKD.

Renin–angiotensin system (RAS) abnormalities are considered important risk factors for the pathogenesis of cyclosporin toxicity [14]. CsA promotes vasoconstriction by stimulating the release of angiotensin II, a potent vasoconstrictor, and upregulates AT1 receptors (AT1-R) [87]. In this study, we evaluated the effect of mild unconjugated hyperbilirubinemia induced by UGT1A1 antisense on mRNA expression of angiotensin II receptor type-1 (AT1-R) to assess the reno-toxic effects of CsA. Our data demonstrated that renal AT1-R was increased in the CsA-treated group compared with the control group. Several investigations have shown that CsA-treated rats have enhanced plasma renin activity [88], Ang II levels [89], and tissue renin synthesis [90]. Furthermore, CsA therapy increases angiotensin-converting enzyme activity (ACE) [91], upregulates AT1-R in vascular and renal tissue [92], and accelerates the vasoconstrictive effects of Ang II [93]. Treatment with UGT1A1 antisense in combination with CsA significantly decreased mRNA expression of renal AT1-R compared with the CsA-treated group. In mice, increasing circulating bilirubin prevents the hypertensive effects of ANG II infusion [94]. Furthermore, hyperbilirubinemia Gunn rats are resistant to the pressor effects of ANG II and have lower systolic blood pressure [95]. The data obtained from the current study strongly suggest that physiologically relevant UCB concentrations downregulated AT1-R mRNA relative expression, which has clear implications for hypertension pathogenesis.

Several tissue markers for kidney injury detect the early onset of CsA nephropathy and enable its progression to be monitored would be extremely useful, minimizing kidney injury progression [96]. These markers included fibronectin [97], kidney injury molecule-1 (KIM-1) [98], and neutrophil gelatinase-associated lipocalin (NGAL) [99]. Fibronectin (FN) is a kidney matrix glycoprotein that connects epithelial cells to the extracellular matrix collagen via integrin [100]. KIM-1 is an undetectable transmembrane glycoprotein excreted in the urine after proximal tubular kidney injury [101]. NGAL is a low-molecular-weight acute-phase protein highly expressed in injured epithelial cells and freely filtered by the glomerulus [102]. Our current findings show that fibronectin, KIM-1, and NGAL mRNA relative expressions were increased in kidney tissues of CsA-treated mice compared with the control group. The progression of chronic kidney injury is associated with increased fibronectin expression in renal tissue [103]. Elevated expression of renal fibronectin expression has also been linked to the development of interstitial fibrosis [104].

Renal KIM-1 levels were found to be significantly higher at all stages of CsA-induced nephrotoxicity, indicating that CsA-induced tubular injury occurs early, before interstitial fibrosis, and is a continuous process throughout the drug exposure period [105]. Furthermore, the expression level of NGAL in renal tubules is rapidly upregulated when they are injured [106]. CsA-induced renal injury was found to increase the expression of Kim-1 and NGAL, indicating that CsA damages renal tubular cells [107]. The present study found that a physiologically increased UCB was more effective than the natural CH in attenuating kidney injury induced by CsA therapy. Mice with mild hyperbilirubinemia had significantly lower mRNA relative expression in the renal Kim-1 and NGAL genes compared with the CsA-treated group. The reno-protective effect of bilirubin after cyclosporine A exposure was demonstrated in rats by a significant reduction in urine Kim-1 and a decreased tendency of urine NGAL concentration, indicating that bilirubin aids in the prevention of epithelial cell injury [108].

CsA treatment caused significant changes in the histological structure of kidney tissue, which corresponded to changes in biochemical, oxidative, and molecular markers observed in our study. The histological findings of H&E-stained kidney sections indicated that CsA treatment induced glomerular hypertrophy and enlarged Bowman’s space with mesangial proliferation, tubular degeneration in the cortex, tubular degeneration, and necrosis in the medulla. Excessive, bluish-colored collagen deposition in interstitial tissue in the cortex and medulla was seen in Masson trichrome-stained sections. PAS-stained kidney sections revealed glomerular enlargement and increased thickness of the PAS-positive glomerular basement membrane. The histopathological changes obtained from the present study were consistent with that of Fetouh and Hegazy, [109], who discovered some histopathological changes in kidney tissue after CsA treatment, such as glomeruli shrinkage, Bowman’s space expansion, Bowman’s capsule thickening, as well as renal tubule cells vacuolization and basement membrane thickening. Rezzani [110] discovered extensive tubular degeneration in his study, which is consistent with our findings.

## 5. Conclusions

In summary, it has been discovered that antisense morpholinos targeting hepatic UGT1A1 generate physiological unconjugated hyperbilirubinemia serum. Our findings support the potential protective effect and the underlying mechanisms of physiological UC hyperbilirubinemia in a mice model of cyclosporine A-induced kidney disease. Physiological UCB protects against kidney disease and restores kidney functions through various mechanisms, including antioxidant, oxidative stress inhibition, anti-inflammatory, vascular endothelial protection, and hormonal action by activating nuclear hormone receptors (PPAR-α). Moreover, it significantly downregulated mRNA expression of NF-kB, ETA-R, iNOS, AT1-R, cFn, Kim-1, and NGAL in the kidney tissue and alleviated CsA-induced kidney histological changes in CsA-treated mice and via activation of PPAR-α, which regulates the expression of multiple genes implicated in kidney disease, and normalization of altered histological changes in the kidney. As a result, UC hyperbilirubinemia within a physiological range is a proper pharmacologic strategy for preventing CsA-induced kidney injury without the dangers of drug-related side effects. These findings may help to explain the link between UCB and protection against CKD outcomes.

## Data Availability

On reasonable request, the corresponding author will provide the datasets created and/or analyzed during the current work.

## Supplementary Materials

The following supporting information can be downloaded at: https://www.mdpi.com/article/10.3390/metabo12100999/s1, Figure S1: Masson trichrome-stained kidney sections; Figure S2: PAS-stained kidney sections; Table S1. Primer sequences used for the target genes and control gene GAPDH in mice.

## Abbreviations

ACE	Angiotensin-converting enzyme
AF	Atherosclerotic factor
ARF	Acute renal failure
AT1-R	Angiotensinogen type-1 receptor
BVs	Blood vessels
bp	Base pair
CAT	Catalase
CB	Conjugated bilirubin
cFn	Cellular fibronectin
CHD	Coronary heart disease
CKD	Chronic kidney disease
CVD	Cardiovascular disease
CNIs	Calcineurin inhibitors
CsA	Cyclosporine A
CH	Chitosan
eGFR	Estimated glomerular filtration rate
ESRD	End-stage renal disease
ET-1	Endothelin-1
ETA-R	Endothelin type A-receptor
FAS	Fatty acid synthase
G	Glomeruli:
GAPDH	Glyceraldehyde-3 phosphate dehydrogenase
GFR	Glomerular filtration rate
GSH	Reduced glutathione
GST	Glutathione S-transferase
GPx	Glutathione peroxidase
H&E	Hematoxylin & Eosin
IF_K_B	Inhibitory factor _k_B
INOS	Inducible nitric oxide synthase
Kim1	Kidney injury molecule 1
LSD	Least significant difference
MDA	Malondialdehyde
MERC	Medical experimental research center
MT	Medullary tubules
NF-kB	Nuclear factor kappa B
NGAL	Neutrophil gelatinase-associated lipocalin
NO	Nitric oxide
PRAR-α	Peroxisome proliferator-activated receptor alpha
PAS	Periodic acid–Schiff
RCT	Reverse cholesterol transfer
ROS	Reactive oxygen species
RT	Reverse transcription
RT-PCR	Real-time-polymerase chain reaction
SE	Standard error
SiRNA	Small interfering RNA
SOD	Super oxide dismutase
SPPARM	Selective PPAR modulator
T	Tubules
TB	Total bilirubin
U	Urea:
UCB	Unconjugated bilirubin
UGT1A1	Uridine diphosphate-glucuronyl transferase1A1
