# Supplementary material for: The Potential Protective Effect and Underlying Mechanisms of Physiological Unconjugated Hyperbilirubinemia Mediated by UGT1A1 Antisense Oligonucleotide Therapy in a Mouse Model of Cyclosporine A-Induced Chronic Kidney Disease
Source: Metabolites. 2022 Oct 20;12(10):999. doi: 10.3390/metabo12100999 (PMC9612357; doi:10.3390/metabo12100999)
Supplement: Supplementary file 1 [file metabolites-12-00999-s001.zip › metabolites-1890106-supplementary.pdf]

Article

The Potential Protective Effect and Underlying Mechanisms of Physiological Unconjugated Hyperbilirubinemia Mediated by UGT1A1 Antisense Oligonucleotide Therapy in a Mouse Model of Cyclosporine A-Induced Chronic Kidney Disease

Basma H. Marghani [1,2,*], Mohamed El-Adl [3], Ahmed I. Ateya [4], Basma H. Othman [5], Heba I. Ghamry [6], Mustafa Shukry [7,*], Mohamed Mohamed Soliman [8] and Mohamed Abdo Rizk [9]

1 Department of Physiology, Faculty of Veterinary Medicine, Mansoura University, Mansoura 35516, Egypt
2 Department of Biochemistry, Physiology, and Pharmacology, Faculty of Veterinary Medicine, King Salman International University, South of Sinaa 46612, Egypt
3 Department of Biochemistry, Faculty of Veterinary Medicine, Mansoura University, Mansoura 35516, Egypt
4 Department of Husbandry & Development of Animal Wealth, Faculty of Veterinary Medicine, Mansoura University, Mansoura 35516, Egypt
5 Medical Research Center, Faculty of Medicine, Mansoura University, Mansoura 35516, Egypt
6 Department of Home Economics, College of Home Economics, King Khalid University, P.O. Box 960, Abha 61421, Saudi Arabia
7 Department of Physiology, Faculty of Veterinary Medicine, Kafrelsheikh University, Kafrelsheikh 33516, Egypt
8 Department of Biochemistry, Faculty of Veterinary Medicine, Benha University, Benha 13518, Egypt
9 Department of Internal Medicine and Infectious Diseases, Faculty of Veterinary Medicine, Mansoura University, Mansoura 35516, Egypt
* Correspondence: basmahamed@mans.edu.eg (B.H.M.); mostafa.ataa@vet.kfs.edu.eg (M.S.)

Supplementary data

Supplementary Figure S1: Masson trichrome-stained kidney sections

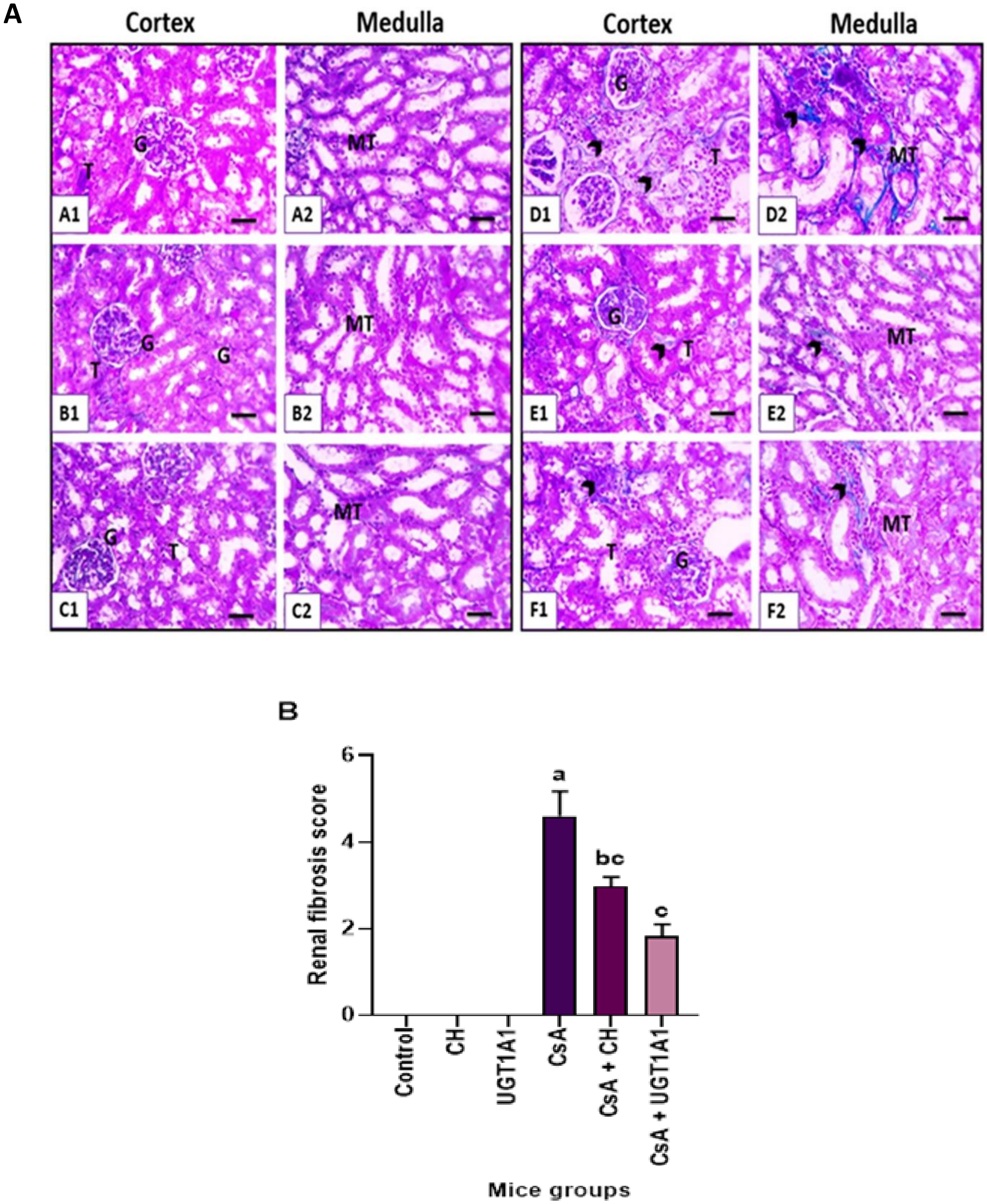

Figure S1: A) Photomicrograph of kidney sections in different experimental groups (Masson trichrome; X: 400). No collagen deposition in interstitial tissue neither in cortex

nor in medulla in kidney sections of the control group (A1, A2), CH-treated group (B1, B2), and UGT1A1 antisense oligonucleotide-treated group (C1, C2). Excessive bluish stained collagen deposition in interstitial tissue (arrowheads) in cortex and medulla in kidney sections of CsA-treated group (D1, D2). Markedly decreased bluish stained collagen depo-sition in interstitial tissue (arrowheads) in cortex and medulla in kidney sections of CsA + CH-treated group (E1, E2). Moderately decreased bluish stained collagen deposition in in-terstitial tissue (arrowheads) in cortex and medulla in kidney sections of CsA + UGT1A1 antisense oligonucleotide-treated group (F1, F2). Glomeruli (G) and tubules (T), medullary tubules (MT). B) Semi-quantitative analysis of renal tissue lesions based on the highest % of fields affected with renal interstitial fibrosis (Trichrome positive area %) expressed as a score (0-5) in different experimental groups. Values are expressed as mean ± SEM (n = 8). Means of different letters are different statistically ($P < 0.05$). (ANOVA; Duncan's post hoc analysis).

Supplementary Figure S2: PAS-stained kidney sections

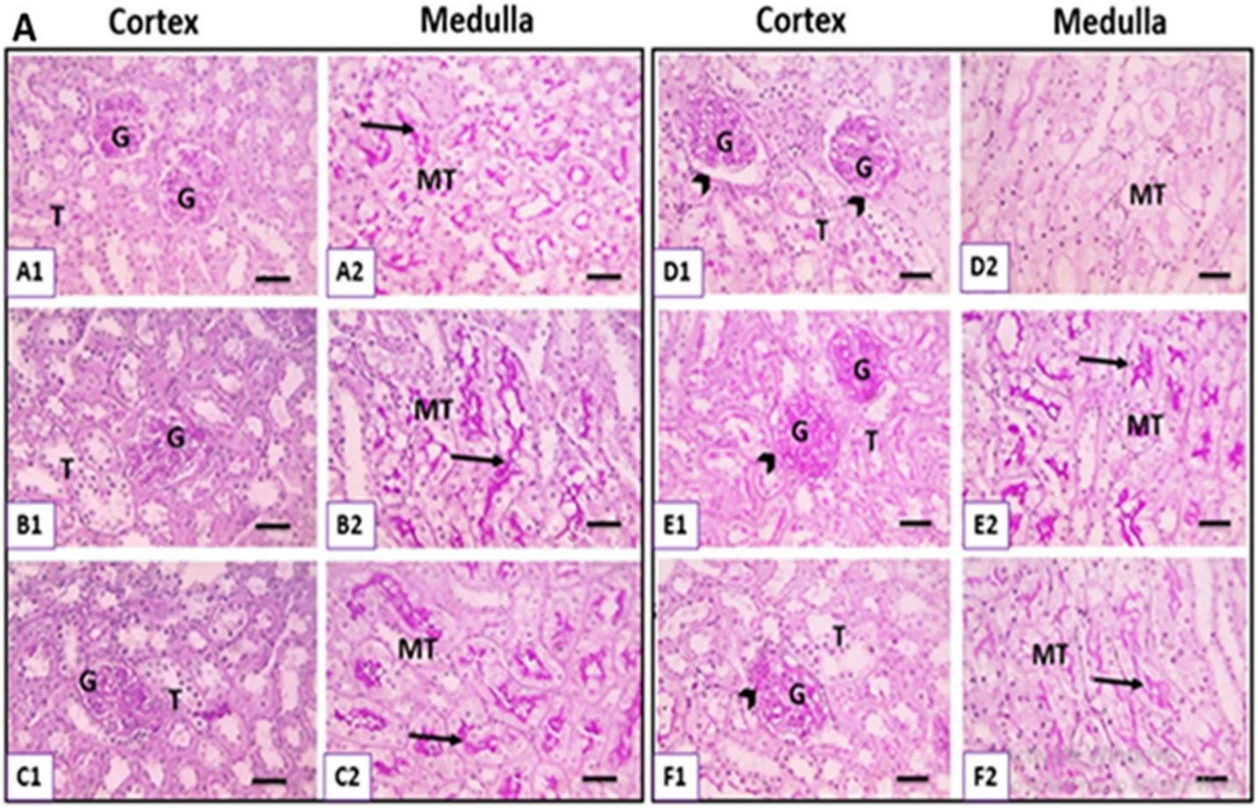

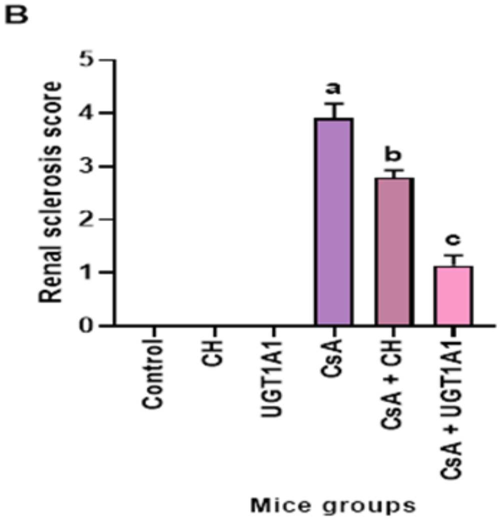

Figure S2: A) Photomicrograph of kidney sections in different experimental groups (PAS; X: 400). Normal structure of cortical glomeruli (G) and tubules (T) besides normal medullary tubules (MT) in kidney sections of the control group (A1, A2), CH-treated

group (B1, B2), and UGT1A1 antisense oligonucleotide-treated group (C1, C2) where the nucleus stained blue, glomerular basement membrane and MT brush borders stained purple (arrows). Glomerular swelling, increased thickness of PAS-positive glomerular basement membrane (arrowheads) with markedly increased renal capsular space, and absent MT brush borders in kidney sections of CsA-treated group (D1, D2). Markedly decreased renal capsular space (arrowhead) with retained PAS positive MT brush borders (arrow) in kidney sections of CsA + CH-treated group (E1, E2). Very mildly thickened glomerular basement membrane (arrowheads) and partially retained PAS positive MT brush borders (arrow) in kidney sections of CsA + UGT1A1 antisense oligonucleotide-treated group (F1, F2). Glomeruli (G) and tubules (T), medullary tubules (MT). B) Semi-quantitative analysis of renal tissue lesions based on the highest % of fields affected with sclerosis (PAS-positive area %) in the cortex and medulla expressed as a score (0-4) in different experimental groups. Values are expressed as mean ± SEM (n = 8). Means of different letters are different statistically (P < 0.05). (ANOVA; Duncan's post hoc analysis).

Supplementary Table S1. Primer sequences used for the target genes and control gene GAPDH in mice.

Primer*	Forward	Reverse	Accession-numbers
PPAR-α	5'-GTGGCTGCTATAATTTGCTGTG-3'	5'-GGAGTTTTGGGAAGAGAAAGGT-3'	XM_030248424.2
NF-κB	5'-CAGGACCAGGAACAGTTCGAA-3'	5'-CCAGGTTCTGGAAGCTATGGAT-3'	AF199371.2
ETA-R	5'-TTGACCTCCCCATCAACGTG-3'	5'-AGCACAGAGGTTCAAGACGG-3'	NM_010332.2
iNOS	5'-TAGAGGAACATCTGGCCAGG-3'	5'-TGGACCACTGGATCCTGCCG-3'	NM_001313922.1
AT1-R	5'-CACCTATGTAA-GATCGCTTC-3'	5'-GCACAATCGCCATAATTATCC-3'	XM_006516534.3
cFn	5′-CTGAACCAGCCTACAGATGAC-3'	5'-CATTTTCTCCCTGCCGATCC-3'	NM_001276413.1
Kim-1	5'-ACGGCTCTCTCCTAACTGGT -3'	5'-CCACCACCCCCTTTACTTCC-3'	NM_001166632.1

NGAL	5'-GGCCAGTTCACTCTGGGAAA-3'	5'-TGGCGAACTGGTTGTAGTCC-3'	NM_008491.1
GAPDH	5'-GCATCTTCTTGTGCAGTGCC -3'	5'-TACGGCCAAATCCGTTCACA -3'	AY618199.1

* PPAR-α, peroxisome proliferator-activated receptor-alpha; NF-κB, nuclear factor kappa B; ETA-R, Endothelin type A-receptor; iNOS, inducible nitric oxide synthase; AT1-R, Angiotensin type1-receptor; cFn, cellular fibronectin; Kim-1, Kidney injury molecule-1; NGAL, neutrophil gelatinase-associated lipocalin; and GAPDH, glyceraldehyde-3-phosphate dehydrogenase.
